# Glioblastoma Immune Landscape and the Potential of New Immunotherapies

**DOI:** 10.3389/fimmu.2020.585616

**Published:** 2020-10-14

**Authors:** Thomas Daubon, Audrey Hemadou, Irati Romero Garmendia, Maya Saleh

**Affiliations:** ^1^University of Bordeaux, CNRS, Institut de Biochimie et Génétique Cellulaires (IBGC), UMR 5095, Bordeaux, France; ^2^University of Bordeaux, CNRS, ImmunoConcEpT, UMR 5164, Bordeaux, France; ^3^Department of Medicine, McGill University, Montreal, QC, Canada

**Keywords:** glioblastoma, immune response, macrophage, immunotherapy, CART-T cell

## Abstract

Glioblastoma (GBM) are the most common tumors of the central nervous system and among the deadliest cancers in adults. GBM overall survival has not improved over the last decade despite optimization of therapeutic standard-of-care. While immune checkpoint inhibitors (ICI) have revolutionized cancer care, they unfortunately have little therapeutic success in GBM. Here, we elaborate on normal brain and GBM-associated immune landscapes. We describe the role of microglia and tumor-associated macrophages (TAMs) in immune suppression and highlight the impact of energy metabolism in immune evasion. We also describe the challenges and opportunities of immunotherapies in GBM and discuss new avenues based on harnessing the anti-tumor activity of myeloid cells, vaccines, chimeric antigen receptors (CAR)-T and -NK cells, oncolytic viruses, nanocarriers, and combination therapies.

## Preface

The adult human brain is a tissue of vast complexity, composed of multiple cell types defined by their location, function, or molecular characteristics. Five main classes of cerebral cells have been described: neurons, astrocytes, oligodendrocytes, endothelial cells, and microglia. Interactions among these cell types orchestrate the structure and function of the brain in electrical signaling, axonal ensheathing, regulation of blood flow, metabolic coupling and immune surveillance. For instance, astrocytes which are key effectors of the brain's energy metabolism, convert glucose into lactate, which is delivered to neurons and retro-converted into pyruvate to fuel the Krebs cycle (1). The neurovascular unit (NVU), which encompasses the blood-brain barrier (BBB), is a functional physiological unit that regulates the blood/cerebral parenchyma interface. It is composed of endothelial cells, smooth muscle cells, pericytes, astrocytes, microglia and neurons. The NVU governs brain homeostasis, controlling cerebral perfusion and protecting from potential pathogens or toxins present in the blood. The NVU is significantly altered in CNS malignancy, especially in glioblastoma (GBM), which are grade IV malignant glioma that are highly vascularized with dense tortuous and leaky blood vessels, permitting massive immune cell infiltration in the tumor core. GBMs are mainly derived from neural stem cells, differentiating into astrocytic or neuronal lineages. This cancer is one of the deadliest types in humans, with an average survival time of <15 months upon diagnosis. Even with the standard-of-care treatment, consisting of surgical resection when possible, followed by radiation and chemotherapy with the drug Temozolomide (TMZ), the estimated recurrence rate is more than 90%. Recurrence is mostly caused by the regrowth of highly invasive cells that spread out of the tumor core, partially due to its hypoxic and acidic environment (2), and are therefore not removed by surgical resection. The long-standing assumption that GBM tumors were clonal masses with identical molecular characteristics have recently been challenged. Indeed, tumor single cell transcriptomics have identified several GBM cellular states with notable plasticity modulated by the tumor microenvironment (3, 4).

## Immune Mechanisms of the Healthy Central Nervous System (CNS)

**Figure 1:**
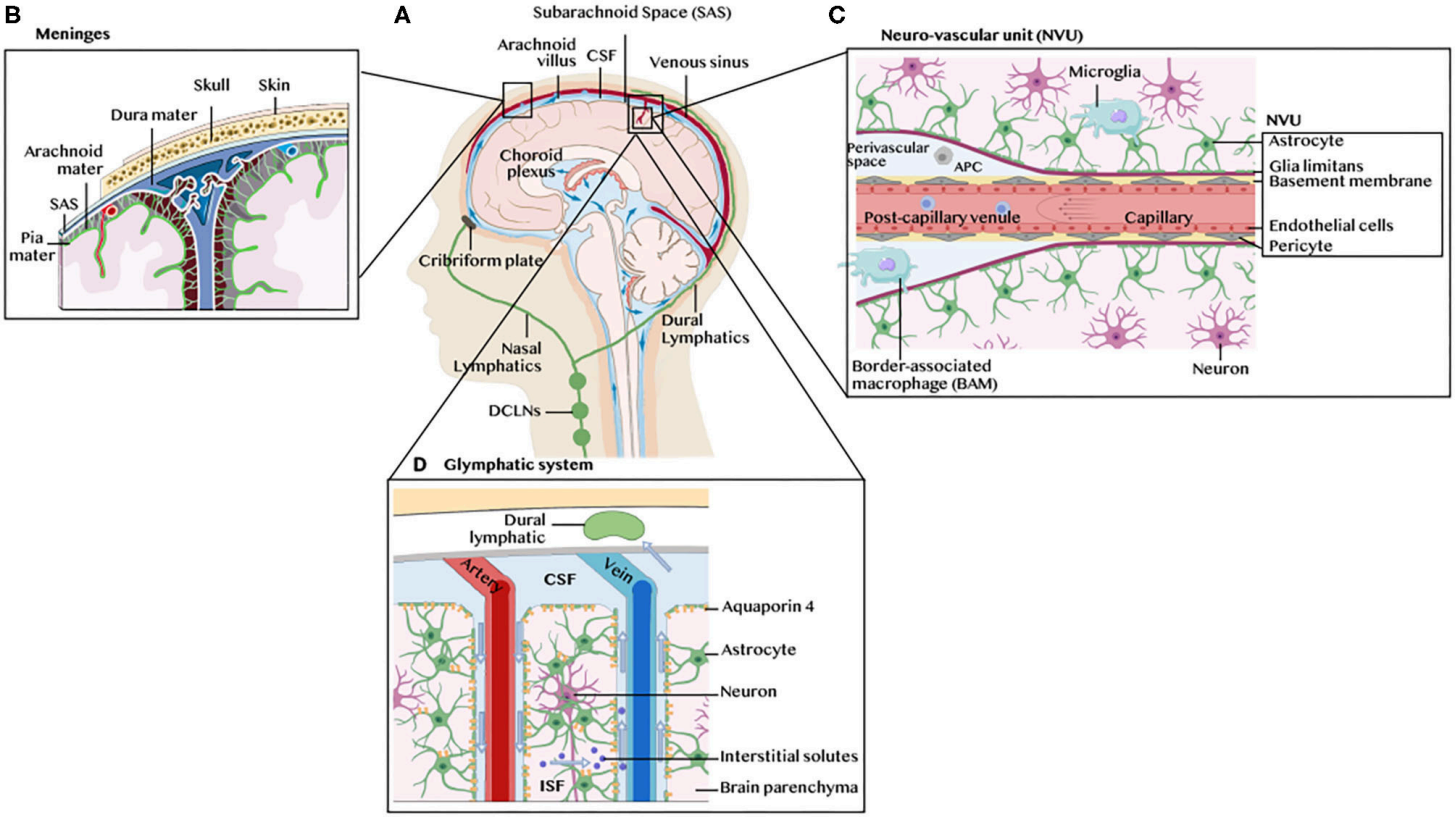
Architecture of the CNS immune system. **(A)** Schematic illustration of the human brain anatomy namely the brain parenchyma, choroid plexus, ventricles, cerebrospinal fluid (CSF), meninges, dural and nasal lymphatics and the deep cervical lymph nodes (DCLNs). **(B)** The meninges. These are three membranes that envelop the brain, namely the dura mater, the arachnoid membrane and the pia mater. **(C)** The neurovascular unit (NVU), blood brain barrier (BBB) and perivascular space. The glia limitans formed by astrocytes end feet ensheath the capillary basement membrane and its pericytes. The perivascular space contains microglia-like perivascular macrophages also dubbed border-associated macrophages (BAMs) and antigen-presenting cells (APC). Microglia are found in the brain parenchyma. **(D)** The glymphatic system. The CSF enters the brain parenchyma through aquaporin 4, water channels on the end-feet of astrocytes surrounding the vasculature, and communicates with the interstitial fluid (ISF) carrying solutes and small antigens through the glymphatic system, a network of perivascular channels formed by astroglia for waste elimination.

Prior to delving into the immune landscape and immunosuppressive mechanisms of GBM, we briefly overview the architecture of the CNS immune system under physiological conditions, highlighting its unique lymphatic drainage system, immune cell populations and leukocyte trafficking (Figure 1A). Anatomically, the brain parenchyma is surrounded by the meninges, a series of three membranes under the skull, namely the dura mater, the arachnoid membrane and the pia mater (Figure 1B). The brain bathes in cerebrospinal fluid (CSF), generated at the blood-CSF barrier, by epithelial cells of the choroid plexus, through diffusion, pinocytosis and active transport from arterial blood in fenestrated capillaries (Figure 1C). The CSF flows around the brain four ventricles into the subarachnoid space (SAS) in a unidirectional flux through the action of cilia on the choroid plexus and ependymal cells that line the ventricles. It enters the brain parenchyma through aquaporin 4, water channels on the end-feet of astrocytes surrounding the vasculature, and communicates with the interstitial fluid (ISF) through the glymphatic system, a network of perivascular channels formed by astroglia for waste elimination (5). The CSF is reabsorbed by the venous blood in venous sinuses at arachnoid villi. Such turnover occurs three to twelve times daily suggesting that the CSF is an immunologically active fluid. Indeed, the CSF drains trafficking leukocytes to the deep cervical lymph nodes (DCLNs) via the newly discovered meningeal lymphatic vessels in the dura mater (6, 7), or by channeling along cranial nerves through the cribriform plate to the nasal mucosa where it accesses its afferent lymphatics. The ISF, which carries parenchymal solutes and small soluble antigens but not parenchymal immune cells, reaches the DCLNs by channeling along the tight space of the basement membrane lining the walls of cerebral capillaries and arteries. The blood supply of the brain enters through capillaries and post-capillary venules, that push the pia mater in the SAS to form perivascular spaces (Virchow-Robin spaces). The brain vasculature is ensheathed by the BBB (Figure 1D) formed by endothelial cells connected by complex tight junctions and pericytes in the capillary basement membrane, and surrounded by the pia mater, the subpial space and the glia limitans, a thin membrane barrier at the parenchymal basement membrane formed by astrocyte foot processes.

The CNS has long been considered as a site of immune privilege. This was based on earlier findings that transplanted tissue grafts in the brain parenchyma elicit slow adaptive immune responses and are not readily rejected (8), and on the presumed lack of lymphatic vessels. Further, a paucity of innate immune responses to pathogen- or danger-associated molecular patterns (PAMPs and DAMPs) has been reported (9, 10). However, mounting evidence challenge this notion and demonstrate active immunosurveillance in the healthy CNS (11). Together with the discovery of a dural meningeal lymphatic system (6, 7), several studies have shown that unlike the brain parenchyma, the cerebral ventricles elicit immune responses leading to graft rejection (12, 13). Thus, the CNS exhibits compartment-specific immunity regulated by leukocyte entry across endothelial, epithelial and glial cell layers of the blood-brain and blood-CSF barriers. These barriers segregate the parenchyma from the peripheral immune system at steady state while permitting immune communications in the CSF-filled SAS and ventricular space. Such compartmentalization is also reflected by spatially and functionally diverse resident immune cell subsets.

The recent use of high-dimensional single cell approaches [e.g., mass cytometry and single cell RNA sequencing (scRNAseq)] in mice (14) and humans (15), along with intravascular leukocyte tracking and fate mapping systems in reporter mice, has uncovered diverse resident immune cells in the healthy CNS and mapped their localization to different CNS compartments. Microglia, which are derived from a yolk sac progenitor, are found exclusively in the brain parenchyma. A distinct subset of embryonically-derived microglia-like macrophages line the meninges, the choroid plexus and the perivascular spaces, and are dubbed border-associated macrophages (BAMs). Microglia and BAMs make up the bulk of the healthy CNS immune cells accounting for ~80% and ~10% of all CNS steady state leukocytes, respectively. Blood-derived monocytes (Ly6C^hi^ and Ly6C^lo^), monocyte-derived cells (MdCs), dendritic cells (DCs) and neutrophils are also present in the healthy CNS, albeit at lower frequencies (<3%) (14). T and B cells, innate lymphocytes (ILCs), natural killer (NK), NKT, eosinophils and mast cells are rare (<1%) but also found at steady state. While microglia and BAMs share several surface markers (CD45^lo^ CD11b^lo^ F4/80^+^ CD64^+^ MeTK^+^ Cx3CR1^+^), they differ in the expression of SIGLEC-H, which is typically found on microglia but not on BAMs. In contrast, the latter express CD206, CD38 and CD88. Both subsets potentially act as antigen-presenting cells (APCs), as they can upregulate, in a context-dependent fashion, the expression of CD11c, MHCII and co-stimulatory molecules. For instance, microglia of the white matter express higher levels of MHCII, CD68 and HLA-DR compared to gray matter microglia, and upregulate pro-inflammatory cytokines such as SPP1 (osteopontin) with age (15). There is little evidence that microglia and BAMs migrate to the periphery to prime T cells. Instead they are thought to maintain tissue homeostasis and to locally re-stimulate T cells. On the other hand, brain DCs traffic to the DCLNs using one of two routes: a specific route involving the rostral migratory stream (16), olfactory bulb, cribriform plate, and nasal mucosal lymphatics or via the dural lymphatics (Figure 1A). At steady state, DC trafficking contributes to CNS immune tolerance by inducing regulatory T cells (T_reg_). Endothelial cells of the meningeal lymphatic vessels are also presumed to maintain brain antigens-reactive T cells in an anergic state (7). Efferent T cells reach the CNS through the choroid plexus or subarachnoid veins and extravasate into the CSF-filled ventricular space and SAS. In the absence of antigen encounter, T cells are eliminated from the CNS by apoptosis or CSF drainage. Cognate antigen recognition on perivascular or leptomeningeal APCs is required for activated T cells to cross the glia limitans into the parenchyma. T cell activation in the brain is often detrimental leading to neuroinflammation and tissue damage. However, this is not always the case, as T cells can mediate neuroprotective effects in response to CNS injury (17).

## Glioblastoma (GBM) Subtypes and Their Associated Immune Landscapes

**Figure 2:**
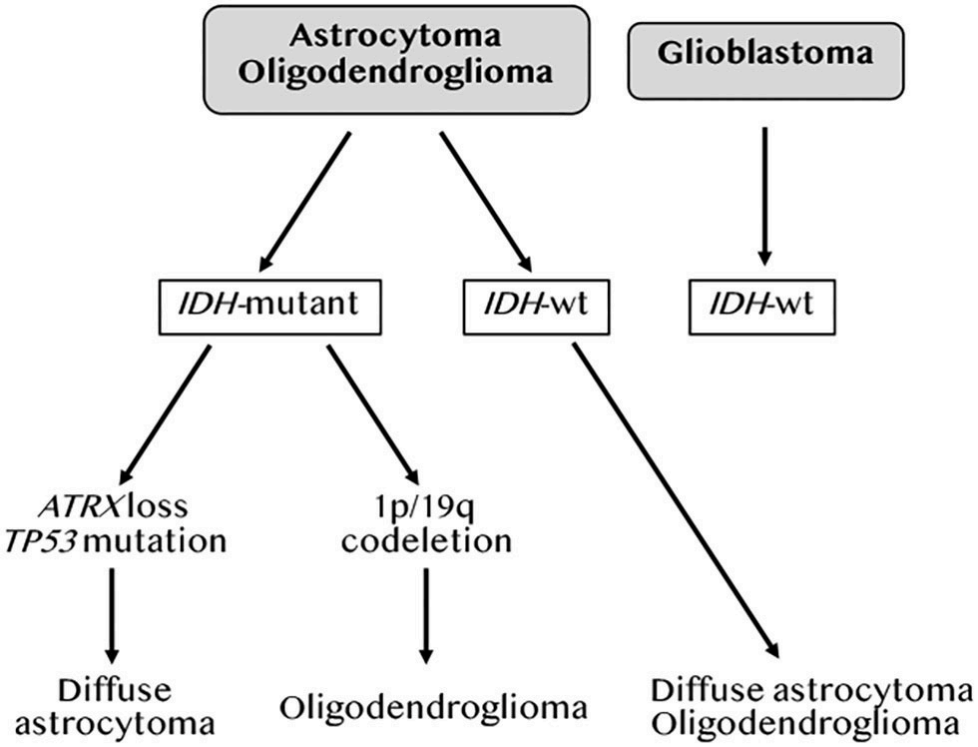
Molecular classification of gliomas. Adapted from the 2016 WHO classification of brain tumors by DeWitt JC et al. (26).

In 2007, the WHO graded CNS tumors based on histological criteria (grade II-IV) (18). In 2010, Verhaak et al. used an unsupervised gene expression analysis of 200 GBM and two normal brain samples to identify four GBM subtypes based on molecular signatures (Table 1). These were referred to as neural (NE), proneural (PN), classical (CL) and mesenchymal (MES) (19). The NE subtype, in which the normal brain samples clustered, was characterized by the expression of neuronal gene markers, and was later shown by the same team to be non-tumor specific (20). The PN subtype, associated with the best median patient survival, had two genomic features, *PDGFRA* alterations and point mutations in *IDH1*, and was characterized by elevated expression of oligodendrocytic and pro-neural development genes. The CL subtype had high rates of *EGFR* gene amplification co-occurring with aberrations in the RB pathway. It exhibited high expression of neural precursors and stem cell markers, and elevated expression of effectors of the Notch and sonic hedgehog pathways. The MES subtype, linked to the least favorable outcome, had predominant *NF1* gene aberrations and *PTEN* mutations. As its name implies, it included an epithelial-to-mesenchymal signature indicative of de-differentiated/trans-differentiated tumors. It also had the highest inflammatory signature with a notable upregulation of genes in the TNF and NF-κB pathways. Several studies from the Cancer Genome Atlas (TCGA) project subsequently defined a core of recurrent driver genomic alterations in GBM, involving *TP53, RB1, NF1, PDGFRA, EGFR, PTEN*, and *CTNND2* (21–24). Genetic alterations in *IDH1* or *IDH2, TERT*, and co-deletion of chromosome arms 1p and 19q (1p/19q codel) were rather found in low grade gliomas (LGG; grades II-III) (23, 25). In 2016, the WHO reclassified CNS tumors to integrate molecular information to the diagnosis criteria (26). This classification divided adult gliomas into three groups: (1) oligodendrogliomas, which harbor IDH mutations and 1p/19q codel, (2) astrocytomas, which are IDH mutant but without the 1p/19q codel, and (3) GBM, which are mostly IDH wild-type (WT) (Figure 2). It also introduced histone 3 K27M mutation as a molecular feature of pediatric diffuse midline glioma (27). More recent integration of results from scRNAseq, *in vivo* single cell lineage tracing and genomic and transcriptomic analyses from TCGA refined the GBM subtypes by identifying four plastic GBM cellular states. These were characterized by six transcriptomic meta-modules and genetic alterations in *EGFR, PDGFRA, CDK4*, and *NF1* (4). Two meta-modules enriched in mesenchymal genes, including hypoxia and glycolysis genes, were referred to as MES1 and MES2, and corresponded to the TCGA-MES subtype in Verhaak et al. (19). An astrocytes-like (AC) module was consistent with the TCGA-CL, and three additional modules referred to as oligodendrocyte progenitor cells-like (OPC) and neural progenitor cells-like (NPC)1 and NPC2, corresponded to the TCGA-PN sub-type (Table 1). Neftel et al. showed, using patient-derived xenografts (PDX) in mice, that tumor cells were able to transit from one cellular state to another, indicative of a plasticity that was modulated by the tumor microenvironment (4).

**Table 1:** GBM molecular classification and associated immune phenotypes.

**Classifier**		**Neural**	**Proneural**	**Classical**	**Mesenchymal**
Genetics^a^		Expression of neuron markers such as NEFL, GABRA1, SYT1 and SLC12A5 Association with GO categories linked to the neuron projection and axon and synaptic transmission	PDGFRA mutations, especially in the Ig-domain Point mutation in IDH1 associated with higher CpG island methylation Focal amplification of the locus at 4q12 harboring PDGFRA High level of PDGFRA expression TP53 mutation Loss of heterozygosity Chromosome 7 amplification paired to loss of chromosome 10 only in 50% of the cases High expression of oligodendrocytic development genes Expression of proneural development genes	Chromosome 7 amplification paired with chromosome 10 loss High level of EGFR amplification High level of EGFR alterations Lack of TP53 mutations Focal 9p21.3 homozygous deletion, targeting CDKN2A High expression of neural precursors and stem cell markers	Focal hemizygous deletion of a region at 17q11.2 Low expression of NF1 Co-mutations of NF1 and PTEN Expression of mesenchymal markers (CHI3L1, CD44, MERKT, YKL40 and MET) High expression of genes implicated in the NFKB and tumor necrosis factor super family pathways (TRADD, RELB, TNFRSF1A) High expression of microglial markers such as CD68 and PTPRC
Immune cell Infiltrates^b^	Tumor core	Macrophages (CD163)	Macrophages (CD163)	Macrophages (CD163) +	Macrophages (CD163) +++
	Tumor edge	Microglia (CD68) ++	Microglia (CD68)	Microglia (CD68) +	Microglia (CD68) +++
	Perivascular area	CD4 T cells ++ CD8 T cells	CD4 T cells CD8 T cells	CD4 T cells + CD8 T cells	CD4 T cells +++ CD8 T cells
Immune markers^c^^,^ ^d^		PD-1	PD-1	IL-12, PD-1	Galectin 3, IL-10, IL-23, TGFβ, PD-L1, CD163, CCR2, CCL-22, CD47, CSF-1, MIC-1, IL-6, CTLA-4, Arginase, CD204, IL1, IL-15, IL-7, CD278, IDO
Re-classification^e^		≪ Healthy brain ≫	Combination of OPC-and NPC-like	AC-like	MES-like
Associated gene mutation with the re-classification ^e^			PDGFRA and CDK4 mutations, respectively	EGFR mutation	NF1 mutation

a *Verhaak RG et al. (19). Cancer Cell 17: 98-110*.

b *Martinez-Lage M et al. (28). Acta Neuropathol Commun 7: 203*.

c *Doucette T et al. (29). Cancer Immunol Res 1: 112-122*.

d *Wang Q et al. (20). Cancer Cell 32: 42-56*.

e *Neftel C et al. (4). Cell 178:835-849*.

The immune landscape of the GBM subtypes was initially explored by transcriptomics (19, 20, 29). These studies confirmed that the MES subtype exhibited elevated expression of pro-inflammatory mediators together with immunosuppressive factors and immune checkpoints (Table 1). CIBERSORT analysis (30) revealed more TAMs, neutrophils and CD4^+^ T cells expression signatures in MES, whereas an activated DCs signature was found in CL (20). Analysis of a separate glioma classification system based on IDH1 mutation status and DNA methylation (31) similarly revealed elevated TAMs and neutrophils signatures in one subgroup of IDH1 wild-type (WT) tumors, that was of the MES profile (20). To reassess these findings at the protein level, Martinez-Lage et al. used an automated immunohistochemistry-based analysis of tissue microarray (TMA) from a cohort of 98 patients to define the immune cell counts in each GBM subtype. Microglia and blood-derived TAMs were the most prevalent cells in all four GBM subtypes, but were highest (>80% of all leukocytes) in the MES subtype. Whereas, CD8^+^ T cell frequencies were similar in all groups, the MES subtype had slightly more CD4^+^ T cells (~1%) (28).

Alternative stratification of GBM based on consensus immunome clusters (CIC) identified two immunologically active GBM clusters (32). These clusters expressed genes associated with cytotoxic T lymphocyte (CTLs) and NK cell activation, such as granzyme B (*GZMB*) and interferon gamma (*IFNG*), and genes linked to feedback inhibitory mechanisms including *FOXP3*, immune checkpoint inhibitors (*CTLA-4, PD-1, TIM3, VISTA*) and their ligands e.g. *PD-L1* and galectin-9 (32). Nevertheless, these CICs did not discriminate patients with respect to survival outcome, potentially due to the low frequencies of CTLs and NKs and the strong immunosuppressive environment mediated by the myeloid compartment. Indeed, GBM tumor-infiltrating lymphocytes (TILs) display an exhausted phenotype (33), and GBM-infiltrating NK cells express reduced levels of activating receptors e.g., NKp30, NKG2D, and DNAX accessory molecule-1 (DNAM-1) (32).

## GBM-Associated Myeloid Cells Diversity, Ontogeny and Tumor Geography

**Figure 3:**
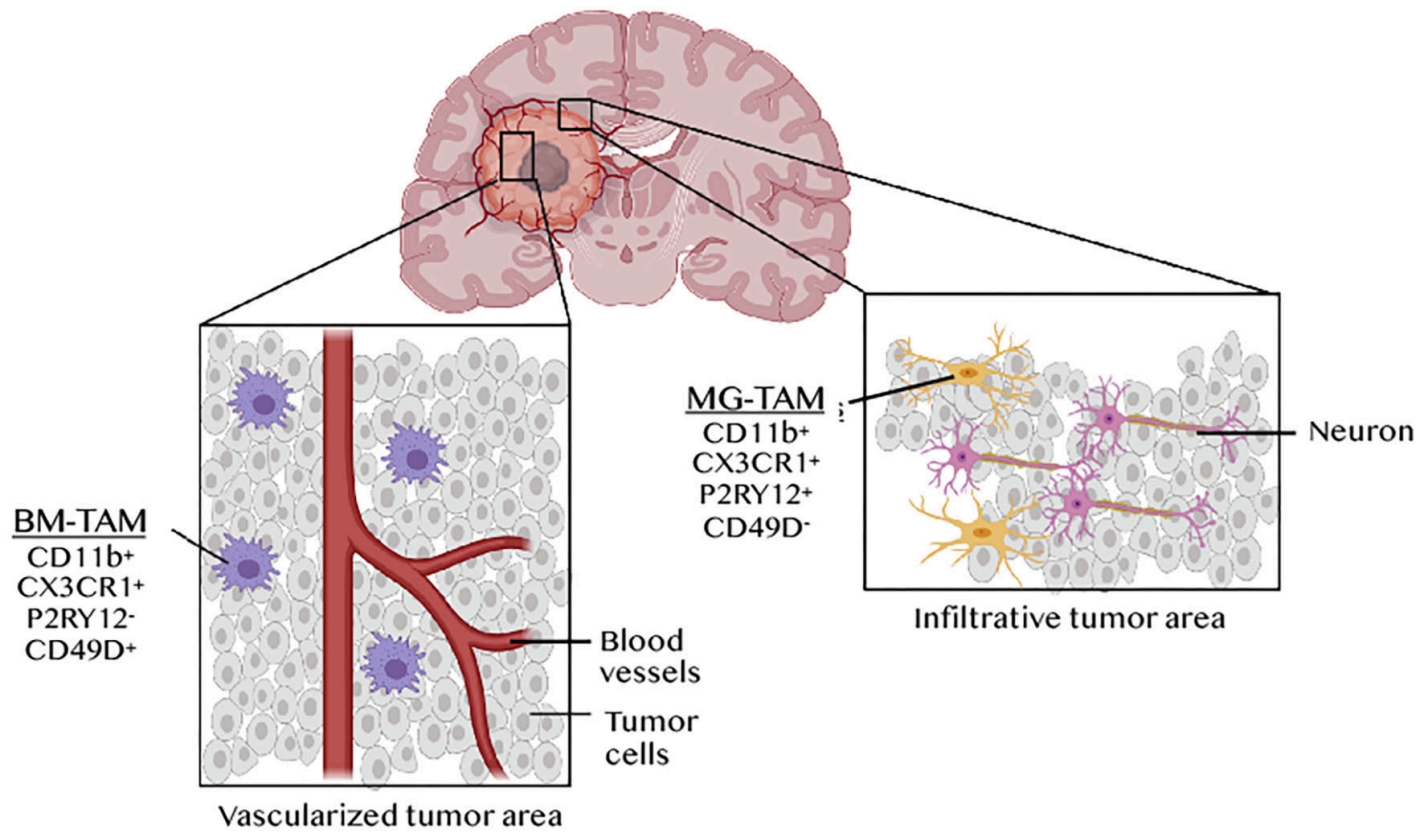
TAM ontogeny and tumor geography in GBM. Tumor-associated macrophages (TAMs) in GBM originates from microglia (MG-TAMs) or from bone marrow-derived monocytes differentiating into macrophages upon recruitment (BM-TAMs). These can be distinguished based on the differential expression of the integrin CD49D on BM-TAMs and of the purinergic receptor P2RY12 on MG-TAMs. BM-TAMs that infiltrate into the tumor core are smaller and less branched than MG-TAMs that are found in the peri-tumoral area.

Myeloid cells are key determinants of tumor progression and patient outcome in several cancers (34), and are being actively pursued as targets of new immunotherapies (35, 36). The predominance and diversity of myeloid cells in GBM has warranted extensive analysis of their phenotypes and functions in this cancer. This is critical for discriminate therapy, as general targeting of macrophages with inhibition of colony stimulating factor 1 receptor (CSF1R) failed to enhance overall survival in recurrent GBM (37). The use of lineage tracing systems in glioma mouse models revealed distinct GBM-associated myeloid cell ontogeny, i.e., TAMs derived from microglia (MG-TAMs) or from hematopoietic stem cells in the bone marrow (BM-TAMs) (38). RNAseq analysis of these subsets highlighted the impact of ontogeny-imposed chromatin states and tumor cues on their functions in tumor growth and response to therapy. For instance, differential resistance to the anti-angiogenesis therapy bevacizumab was reported to be mediated by BM-TAMs (39). ATAC-seq and transcription factor (TF) landscape analysis identified TFs linked to microglia identity [e.g., MEF2 (40)] in MG-TAMs, whereas BM-TAMs were enriched in TFs involved in monocyte to macrophage differentiation, i.e. RUNX, CEBP, PU.1, IRF4 and STAT3. Notably, a RUNX-induced gene, integrin subunit alpha 4 (*Itga4*, also known as Cd49d) was identified as a distinguishing cell surface marker between the two TAM subsets in both mice and humans. It is expressed on BM-TAMs but epigenetically suppressed in microglia and MG-TAMs. Further analysis, using three different scRNAseq platforms, uncovered 66 core genes that distinguish the two TAM lineages (41). CX3CR1, which is commonly used to isolate microglia in mice, is not specific to microglia, since monocytes upregulate its expression as they differentiate in tissues. Instead, the purinergic receptor P2RY12 has recently emerged as a new microglia marker. MG-TAMs are therefore CD11b^+^ CX3CR1^+^ P2RY12^+^CD49D^−^ whereas BM-TAMs are CD11b^+^ CX3CR1^+^ P2RY12^−^ CD49D^+^ (41) (Figure 3). Both TAM subsets display a “non-canonical” state, expressing both M1 and M2 markers. However, BM-TAMs exhibit higher expression of immunosuppressive cytokines and effectors of oxidative metabolism, characteristic of the M2 phenotype (41). Collectively, while several studies confirm a critical role of BM-TAMs in GBM, MG-TAMs are not mere bystanders. A recent report, exploring the efficacy and targets of the phagocytosis checkpoint inhibitor anti-CD47, demonstrated that MG-TAMs are important effectors of glioma cell phagocytosis contributing to overall survival of glioma-bearing mice (42).

RNAseq analysis of distinct anatomically defined tumor regions (e.g., leading edge, infiltrating region, necrotic zone, blood vessels etc.) and *in situ* hybridization for *BIN1* (an MG-TAM marker) or *TGFBI* (a BM-TAM marker), revealed tumor geographic variation in TAM composition. BM-TAMs were enriched near the blood vessels whereas MG-TAMs were found in infiltrated white matter (41). This was confirmed in a glioma model using the Cx3cr1^GFP^;Ccr2^RFP^ reporter mouse, which showed that BM-TAMs, which constituted 85% of the total TAM population, localized in the perivascular areas of the tumor core, whereas MG-TAMs accounting for 15% of all TAMs, were restricted to the peritumoral area (43) (Figure 3). Besides differential gene expression profiles, these two TAM subsets have different morphological and migratory characteristics, as shown by 2-photon microscopy. MG-TAMs are stationary, larger in size and more branched than BMDM-TAMs that are highly mobile and smaller (44). Clinically, BMDM-TAM infiltration correlates with poor patient survival (28, 41).

## TAM Recruitment and Immunosuppressive Mechanisms in GBM

Interleukin (IL)-6, produced by vascular endothelial cells and TAMs, has been implicated in several pro-tumoral processes in GBM: (1) it contributes to the disruption of the BBB by downregulating intercellular tight junction proteins on endothelial cells (45). Concordantly, endothelial cell-specific deletion of IL-6 prevented glioma growth and improved mouse survival (46); (2) it reinforces GBM metabolic dependence on aerobic glycolysis (47), as discussed below; and (3) it promotes the recruitment of macrophages through the induction of CCL5/CXCL5 and favors their alternative activation through PPARγ/HIF-2α signaling (46). The CCL2-CCR2 pathway is equally important for BM-TAM recruitment. Glioma cells instruct this pathway through indoleamine 2,3-deoxygenase (IDO)-dependent production of kynurenine (KYN), a metabolite that triggers CCR2 upregulation through aryl hydrocarbon receptor (AHR). Myeloid-specific deletion of AHR in mice blunted BM-TAMs glioma infiltration. In humans, the KYN-AHR pathway is upregulated in GBM and is associated with an unfavorable outcome (48). A direct correlate has been established between loss of *PTEN* and BM-TAM recruitment via lysyl oxidase (LOX), a macrophage chemoattractant that signals through the β1 integrin (ITGB1)-PYK2 pathway. Concordantly, YAP1, LOX and β1 integrin are elevated in GBM, and are associated with reduced overall survival. LOX-elicited TAMs infiltrate the tumor microenvironment and support glioma growth via SPP1 (osteopontin), which inhibits glioma cell apoptosis, promotes angiogenesis and sustains the TAM tolerogenic phenotype by signaling through the Integrin αvβ5 (49, 50).

GBM and other brain tumors are notorious for eliciting local and systemic immunosuppression, mediated in great part by TAMs. TAM-derived TGFβ was initially considered as a key inducer of systemic immune tolerance (51). However, targeting this immunosuppressive cytokine alone did not impact the survival of mice bearing brain tumors (52), implicating additional mechanisms. The expression of PD-L1 on circulating monocytes and BM-TAMs might similarly trigger systemic immunosuppression, through a feed forward mechanism involving IL-10 (53). Beyond soluble immunosuppressive cytokines, direct cell-cell contacts, e.g., through PD-L1 (54), tolerogenic HLA molecules (55) and the apoptosis-inducing receptor Fas (56) contribute to immune escape. A recent study reported a role of tumor-associated glycosylation in local and systemic immunosuppression (57). This was mediated through a direct interaction between O-linked glycans on glioma cells with their receptor, Macrophage Galactose-type Lectin (MGL), on TAMs leading to immunosuppression signaling. Of note, the current GBM standard of care often prescribes dexamethasone to alleviate cerebral edema. This immunosuppressive corticosteroid further contributes to the GBM immunosuppressive environment, interfering with anti-tumor immunity and presenting a challenge for the future of immunotherapies in this cancer.

## Metabolic Remodeling of the GBM Tumor Microenvironment

Hypoxia and necrosis are well-known features of GBM. HIF-1α, stabilized by the inhibition of prolyl hydroxylase (PHD) activity in hypoxia, is a transcription factor that modifies the expression of thousands of genes, notably effectors of glycolysis and lactic fermentation. The expression of glucose transporters (GLUT1), glycolytic enzymes (PDK1, Hexokinase or PKM2), and lactate dehydrogenase A (LDHA) help in replenishing NAD^+^ to support the glycolytic process. Monocarboxylate transporter (MCT)4 expression is also increased following stabilization of HIF-1α, leading to passive release of lactate out of the cells (58). Production of H^+^ happens during glycolysis, lactic fermentation, but also during respiration when CO2 is hydrated into HCO3^−^ and H^+^ ions by carbonic anhydrases (CAs). H^+^ ions efflux from the cytoplasm via H^+^ ATPases and Na^+^/H^+^ exchangers (NHEs) leads to a decrease in the extracellular pH_e_. Tumor acidosis promotes cancer cell invasion through cytoskeletal remodeling, but also by modulating the activity of immune cells in the tumor microenvironment. For instance, LDHA-mediated production of lactic acid was shown to blunt the cytotoxic activity of CTLs and NK cells in melanoma through inhibition of NFAT expression (59). This supports previous findings demonstrating that lactate accumulation in T cells, due to decreased efflux via MCT1 (which controls lactate shuttling in a gradient dependent manner), blunted CTL activity (60). TAMs reinforce GBM metabolic shift to aerobic glycolysis through IL-6 that enhances the activity of phosphoglycerate kinase 1 (PGK1) by promoting its phosphorylation (47).

Glioma cells also display a high dependence on amino acid metabolism accompanied by an elevated uptake of branched chain amino acids (BCAA). Through the overexpression of branched chain amino acid transaminase 1 (BCAT1), glioma tumors excrete elevated levels of branched-chain ketoacids (BCKA) through MCT1. Which influx into TAMs and blunt their phagocytic activity (61). GBM TAMs were also shown to drive T cell dysfunction through elevated expression of the ectonucleosidase CD39 that, together with CD73, induces the production of the immunosuppressive metabolite adenosine (48).

## The Future of Immunotherapies in GBM

### Immune Checkpoint Inhibitors (ICI)

Immune checkpoint inhibitors (ICI) targeting the PD-1 or CTLA-4 pathways have revolutionized cancer therapy in the last decade. However, they have had little clinical benefit in GBM, at the least in the adjuvant setting. The recently published results of the open-label, randomized, phase 3 trial CheckMate-143, which evaluated nivolumab vs. bevacizumab in patients with recurrent GBM were disappointing, as there was no significant difference in median overall survival (mOS) between the two arms (62). The two ongoing phase 3 trials CheckMate-498 and CheckMate-548 evaluating the use of nivolumab in patients with newly-diagnosed GBM, either methylguanine methyltransferase (MGMT)-unmethylated or MGMT-methylated, also failed to meet their primary endpoints, according to an update by Bristol-Myers Squibb. In the neoadjuvant setting, the results are controversial. The anti-PD-1 nivolumab, administered as a neoadjuvant, did not impact patient survival in resectable GBM in a phase 2 clinical trial (63). In contrast, another study reported a survival benefit of the anti-PD-1 pembrolizumab in 35 patients with recurrent and resectable GBM (64). Collectively, the dismal results of ICI in GBM may be due to the poor immunogenicity of GBM tumors. In 2017, the FDA approved the use of the anti-PD-1 pembrolizumab in solid tumors with microsatellite instability high (MSI-H) or mismatch repair deficiency (dMMR) tumors. This year, it further approved the use of pembrolizumab for the treatment of adult and pediatric patients with non-resectable or metastatic tumor mutation burden-high (TMB-H) solid tumors. dMMR gliomas are rare (65), but earlier results from two case reports showed a response to pembrolizumab in one pediatric (66) and one adult (67) patients. Despite these promising results, a recent study reported that PD-1 blockade did not impact mOS in hypermutated gliomas, consistent with an observed lack of TILs in these cancers (68). However, another study reported significant clinical and radiological responses of nivolumab in two young siblings with biallelic mismatch repair deficiency (66), suggesting that ICI therapy might benefit pediatric GBM with high mutational burden [e.g., with *MSH6* mutations (69)]. It is plausible that treatments that increase mutational burden might synergize with ICI, as has been shown in other cancers (70). Nanoscale immunoconjugates (NICs), which deliver ICIs, covalently attached on a natural biopolymer scaffold, across the BBB using transferrin receptor (TfR)-mediated transcytosis, or via angiopep-2 (AP-2)- LDLR-related protein 1 (LRP1), were shown to outperform free ICIs in increasing TILs and improving survival in a murine glioma model (71). However, this remains to be tested in patients. Alternative immunotherapies for GBM are being explored. These are primarily focused on vaccines, chimeric antigen receptors (CAR)-T cells, oncolytic viruses and strategies that harness the anti-tumor activity of myeloid cells or the use of adipose stem/stromal cells (ASC) and stromal vascular fraction (SVF) injected in the surgical cavity [reviewed in Bateman et al. (72)].

### Vaccines

In the vaccine arena, three phase 3 clinical trials have been completed with different outcomes. ACT IV, a phase III trial evaluating Rindopepimut (also known as CDX-110), a 13-amino acid peptide vaccine targeting EGFRvIII, a constitutively active mutant form of EGFR expressed in ~30% of GBM patients, in combination with TMZ was terminated for futility, as no significant difference in mOS was observed in patients with newly-diagnosed GBM (73). The failure of this approach might be due to heterogenous expression of EGFRvIII within the tumor or loss of its expression leading to clonal outgrowth of resistant cells. A second phase III trial that evaluated an autologous tumor lysate-pulsed DC vaccine (DCVax®-L) in combination with TMZ showed some clinical benefit, reporting longer progression free survival (PFS) and mOS in patients with recurrent GBM (74). However, this is a logistically complicated approach as it requires personalization, apheresis, and DC expansion prior to administration back into patients. A third phase III trial conducted in Japan using personalized peptide vaccination for HLA-24+ recurrent GBM did not meet the primary nor the secondary endpoints (75). More recently, two phase I/Ib trials reported beneficial effects of personalized peptide vaccines. The first, the Glioma Actively Personalized Vaccine Consortium (GAPVAC), employed two sets of personalized peptide vaccines designed according to patients tumor mutations, transcriptomic and immuno-peptidomic profiles, and showed that these vaccines were able to elicit sustained CD8^+^ T cell and CD4^+^ Th1 responses against neoantigens (76). The second, which employed a pool of synthetic long peptides mimicking neoantigens, also reported the generation of poly-functional neoantigen-specific CD4^+^ T cells and CD8^+^ T cells in the periphery and enhanced infiltration of TILs (77). Together, these trials indicate that vaccine approaches are feasible as they elicit anti-tumor immune responses but whether this will translate into clinical benefit, as a monotherapy, requires additional testing.

### CAR-T and CAR-NK Cells

CAR-T cells are patients-derived T cells engineered to express a CAR, which consists of the antigen-recognition region of an antibody fused in tandem with the cytoplasmic domains of the T cell receptor chain CD3ζ and costimulatory receptors (e.g., CD28 and/or 4-1BB). Currently, approved CAR-T cells target CD19 in B cell malignancies. The challenges of this therapy include the identification of tumor-specific or tumor-associated antigens, especially important in solid tumors, circumventing antigen loss, and countering the exhaustion of transferred CAR-T cells, among others. Several trials and pre-clinical studies have been conducted using CAR-T cells in GBM. The first was a case report that used an IL13Rα2-CAR-T cells in one patient. The CAR-T was delivered through repeated infusions in the resected tumor cavity followed by infusions in the ventricular system. This regimen led to the regression of all cranial and spinal tumors accompanied by a notable immune activity in the CSF (78). A first-in-human study including 10 patients with recurrent GBM followed. This study evaluated EGFRvIII-CAR-T cells injected intravenously. While the CAR-T cells expanded in the blood and trafficked to the tumor, the antigen was lost in 5 out 7 patients and the tumor microenvironment exhibited elevated expression of inhibitory molecules and a high frequency of Treg cells (79). Improvement of CAR-T therapy requires the identification of a tumor-associated antigen expressed stably throughout tumor growth and with limited heterogeneity. Chondroitin sulfate proteoglycan 4 (CSPG4) was found to fit this criterion. It is highly expressed in 67% of GBM cells and is sustained by TNF derived from microglia. Intracranial delivery of CSPG4-CAR-T cells was effective *in vivo* in nude mice transplanted with CSPG4-expressing glioma cells or neurospheres (80). Transgenic expression of cytokines, such as IL-15, was also demonstrated as a mean to improve anti-glioma activity of CAR-T cells, as shown with IL13Rα2-CAR-T cells (81). Since the final CAR-T cell product is a mix of CD4^+^ CAR-T and CD8^+^ CAR-T cells, another mean to refine this approach is to characterize the T cell subset that mediates anti-tumor activity. Using orthotopic GBM mouse models and IL13Rα2-CAR-T cells, the CD4^+^ CAR-T cell subset was found to be more effective than the CD8^+^ CAR-T cells, which were rapidly exhausted (82). Co-expression of the IL-8 receptor, CXCR1/CXCR2, was found to enhance CAR-T cells trafficking and persistence in the tumor in a glioma mouse model (83). Engineering EGFRvIII-CAR-T cells to co-express a bispecific T-cell engager (BiTE) against wild-type EGFR was demonstrated to ameliorate this therapy by countering the heterogeneity of EGFRvIII expression (84). A CAR-engineered NK cell targeting both WT EGFR and EGFRvIII mutant, NK-92-EGFR-CAR, was similarly efficient in targeting and killing GBM cells in mice engrafted with patients' mesenchymal GBM stem cells (85). Additional CAR target antigens in GBM include B7-H3 (86, 87), HER2 (88–90) and EphA2 (91), as demonstrated in preclinical studies, and in a phase I dose escalation clinical trial using a HER2-CAR (92). Interestingly, generation of a tri-cistronic transgene encoding three CAR molecules against HER2, EphA2 and IL13Rα2, dubbed universal CAR-T (UCAR), was shown to overcome interpatient heterogeneity and target 100% of tumor cells (93). Another approach to overcome problems of tumor heterogeneity and antigen escape, is a new CAR design employing a toxin as the targeting entity was developed and tested in a murine model of glioma. This is based on GBM cells' affinity to bind chlorotoxin (CLTX) by matrix metalloproteinase-2. (CLTX)-CAR-T cells efficiently limited tumor growth in the absence of off-target effects (94).

### Oncolytic Viruses

Oncolytic viruses (OV) constitute an interesting therapeutic approach in GBM, as besides their lytic activity, they might overcome GBM immunosuppression by stimulating innate immunity. Several types of OVs have been tested including replication-competent viruses such as polio and measles viruses, Herpes simplex viruses (HSV), adenoviruses and retroviruses. Notably, recombinant non-pathogenic polio-rhinovirus chimera (PVSRIPO), which binds the poliovirus receptor CD155 on cancer cells, was evaluated in 61 GBM patients via intra-tumoral injection and was effective in 21% patients who survived past 36 months (95). Replication-deficient adenoviruses, e.g., aglatimagene besadenovec, have also been used as vectors to deliver tumoricidal genes such as the HSV thymidine kinase that converts ganciclovir into a toxic nucleotide analog that poisons infected dividing cells. Two phase II clinical trials evaluated this Adv-tk viro-immunotherapy in GBM and reported improved PFS and OS (96, 97). An oncolytic HSV expressing E-cadherin, a ligand for the inhibitory NK receptor KLRG1, resulted in a better outcome in a glioma mouse model, by inhibiting NK cells and permitting viral spread (98). More recently, a Zika OV was shown to specifically target GBM stem cells (GSCs) rather than neural precursor cells, through a SOX2-Integrin αvβ5 Axis (99), suggesting a potentially superior anti-tumoral activity for brain tumor therapy. A triple combination of anti-CTLA-4, anti-PD-1 and a recombinant oncolytic HSV expressing mouse IL-12 (G47Δ-mIL12) cured most mice in two glioma models. CD4^+^ T cells, CD8^+^ T cells and M1 macrophages mediated this response, highlighting the need for combinatory approaches in future trials (100).

### Macrophage-Based Immunotherapies

Additional promising strategies for GBM immunotherapy include harnessing the anti-tumor activity of myeloid and NK cells. Targeting the phagocytosis checkpoint CD47 using a humanized anti-CD47 antibody, Hu5F9-G4, has shown promise in a glioma PDX mouse model of five aggressive pediatric brain cancers (101). Furthermore, anti-CD47 in combination with TMZ was shown to enhance phagocytosis and promote cytotoxic CD8^+^ T cell priming by stimulating antigen cross-presentation through cGAS-STING activation (102). Members of the *Let-7* micro-RNA family have also been used as a therapeutic tool in a mouse glioma model; they boosted microglial anti-tumor activity by stimulating TLR7 (103). Alternatively, blocking TAM recruitment or polarization has also shown some efficacy in preclinical models. A 4-1BB–osteopontin (OPN) bi-specific aptamer for instance increased median survival by neutralizing macrophage infiltration while co-stimulating effector T cell activity (50). Di-mannose nanocarriers that bind the mannose receptor CD206 on M2 macrophages, used to deliver *in vitro-*transcribed mRNA encoding M1-polarizing transcription factors, were shown to reprogram TAMs and improve survival in different cancer models (ovarian, lung metastasis) including GBM (104).

### Perspectives

**Figure 4:**
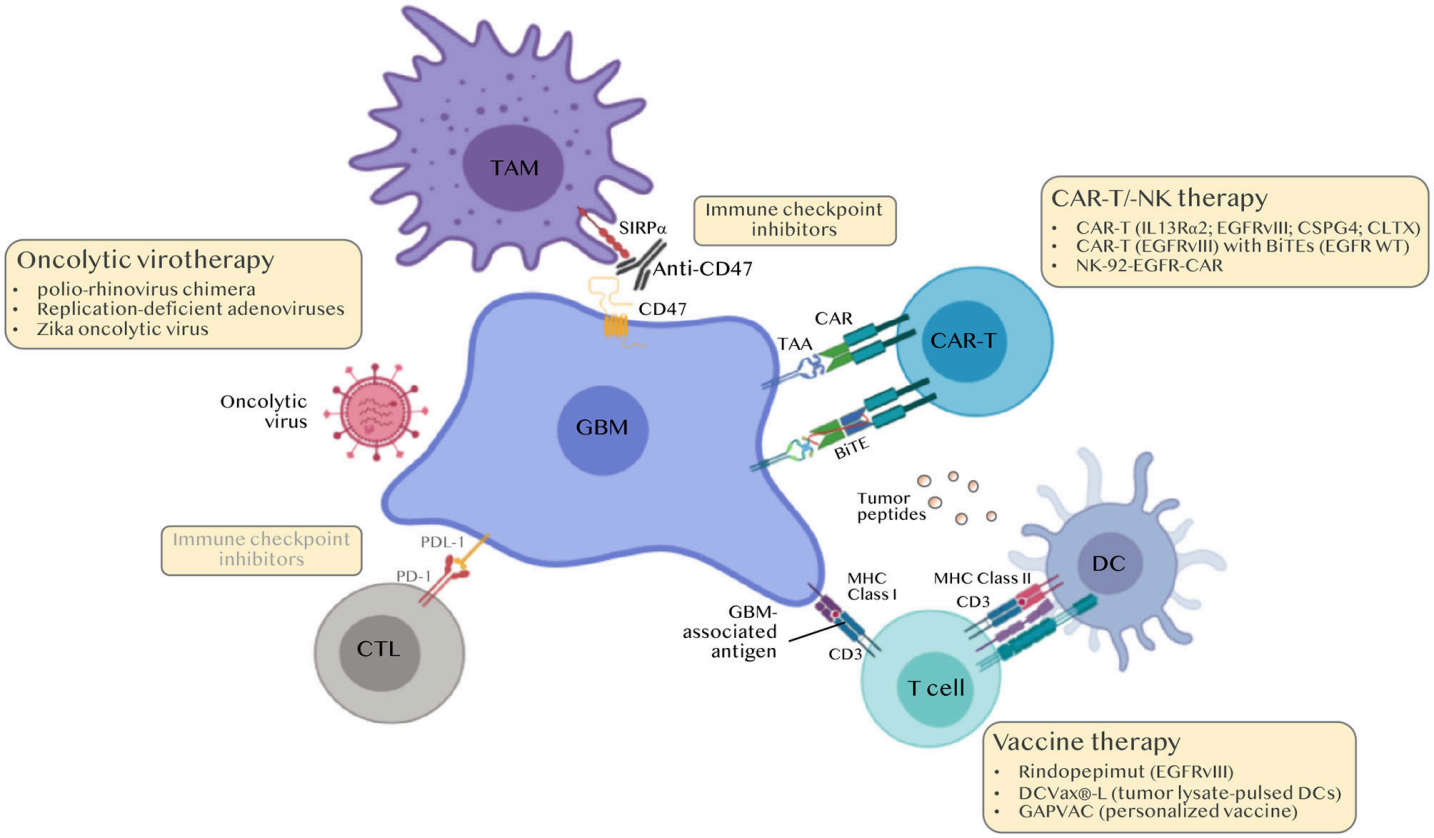
Immunotherapies for the treatment of GBM. Classical immune checkpoint inhibitors (ICI) i.e., anti-PD-1/PDL-1 and anti-CTLA4 were ineffective in GBM. Current approaches include modulating TAMs (anti-CD47 to boost phagocytosis, nano-immunoconjugates to modulate TAM phenotype, aptamers to inhibit TAM recruitment), personalized peptide vaccines, chimeric antigen receptor (CAR)-T and CAR-NK cell approaches and oncolytic viruses. BiTEs, Bi-specific T-cell engagers.

There is a significant need to develop novel GBM immunotherapies. To date, more than 70 clinical trials with the terms GBM and immunotherapy are found in the clinicaltrials.gov webpage, of which 7 are phase III, 31 phase II and 37 phase I trials (Table 2). These trials explore the various strategies described above notably personalized vaccines, adoptive cell transfer therapy and combinations. It is our hope that this endeavor will soon impact patients' lives (Figure 4).

**Table 2:** Clinical trials of immunotherapies for GBM.

**Identifier**	**Study title**	**Interventions**	**Number expected to be enrolled**	**Primary completion**
**Phase III clinical trials**				
NCT04277221	ADCTA for adjuvant immunotherapy in standard treatment of recurrent glioblastoma multiforme (GBM)	Biological: Autologous dendritic cell/tumor antigen, ADCTA	118	December 31, 2022
NCT03548571	Dendritic cell immunotherapy against cancer stem cells in glioblastoma patients receiving standard therapy	Biological: Dendritic cell immunization Drug: Adjuvant temozolomide	60	May 1, 2021
NCT02667587	An investigational immuno-therapy study of temozolomide plus radiation therapy with nivolumab or placebo, for newly diagnosed patients with glioblastoma (GBM, a malignant brain cancer)	Drug: Nivolumab Drug: temozolomide Radiation: Radiotherapy Other: Nivolumab Placebo	693	February 11, 2022
NCT02617589	An investigational immuno-therapy study of nivolumab compared to temozolomide, each given with radiation therapy, for newly-diagnosed patients with glioblastoma (GBM, a malignant brain cancer)	Drug: Nivolumab Drug: Temozolomide Radiation: Radiotherapy	550	January 17, 2019
**Phase II clinical trials**				
NCT04145115	A study testing the effect of immunotherapy (ipilimumab and nivolumab) in patients with recurrent glioblastoma with elevated mutational burden	Biological: Ipilimumab Biological: Nivolumab	37	May 31, 2023
NCT02649582	Adjuvant dendritic cell-immunotherapy plus temozolomide in glioblastoma patients	Biological: Dendritic cell vaccine plus temozolomide chemotherapy	20	December 2020
NCT03927222	Immunotherapy targeted against cytomegalovirus in patients with newly-diagnosed WHO grade IV unmethylated glioma	Biological: Human CMV pp65-LAMP mRNA-pulsed autologous DCs containing GM CSF Drug: Temozolomide Biological: Tetanus-Diphtheria Toxoid (Td) (and 2 more.)	48	December 2023
NCT03916757	V-Boost immunotherapy in glioblastoma multiforme brain cancer	Biological: V-Boost	20	April 15, 2020
NCT03650257	A large-scale research for immunotherapy of glioblastoma with autologous heat shock protein gp96	Biological: gp96 Drug: Temozolomide radiation: Radiotherapy	150	August 20, 2021
NCT03548571	Dendritic cell immunotherapy against cancer stem cells in glioblastoma patients receiving standard therapy	Biological: Dendritic cell immunization Drug: Adjuvant temozolomide	60	May 1, 2021
NCT04013672	Study of pembrolizumab plus SurVaxM for glioblastoma at first recurrence	Drug: Pembrolizumab Drug: SurVaxM Drug: Sargramostim Drug: Montanide ISA 51	51	December 31, 2020
NCT01567202	Study of DC vaccination against glioblastoma	Procedure: Surgery Drug: Chemotherapy Radiation: Radiotherapy (and 2 more.)	100	December 1, 2019
NCT02799238	Autologous lymphoid effector cells specific against tumor (ALECSAT) as add on to standard of care in patients with glioblastoma	Biological: ALECSAT Radiation: Radiotherapy Drug: Temozolomide	62	June 2020
NCT02799238	Cediranib maleate and olaparib compared to bevacizumab in treating patients with recurrent glioblastoma	Biological: Bevacizumab Drug: Cediranib Drug: Cediranib maleate Drug: Olaparib	70	May 31, 2020
NCT02337686	Pembrolizumab in treating patients with recurrent glioblastoma	Other: Laboratory Biomarker Analysis Biological: Pembrolizumab Other: Pharmacological study Procedure: Therapeutic Conventional Surgery	20	December 31, 2020
NCT01174121	Immunotherapy using tumor infiltrating lymphocytes for patients with metastatic cancer	Biological: Young TIL Drug: Aldesleukin Drug: Cyclophosphamide (and 2 more.)	332	December 29, 2023
NCT04225039	Anti-GITR/Anti-PD1/Stereotactic radiosurgery, in recurrent glioblastoma	Drug: INCMGA00012 Drug: INCAGN01876 Drug: SRS Procedure: Brain surgery	32	February 2025
NCT04049669	Pediatric trial of indoximod with chemotherapy and radiation for relapsed brain tumors or newly diagnosed DIPG	Drug: Indoximod Radiation: Partial Radiation Radiation: Full-dose Radiation (and 4 more.)	140	October 2, 2024
NCT03491683	INO-5401 and INO-9012 delivered by electroporation (EP) in COMBINATION WITH cemiplimab (REGN2810) in newly-diagnosed glioblastoma (GBM)	Biological: INO-5401 Biological: INO-9012 Biological: Cemiplimab (and 2 more.)	52	January 18, 2021
NCT03047473	Avelumab in patients with newly diagnosed glioblastoma multiforme	Biological: Avelumab	30	September 2022
NCT03174197	Atezolizumab in combination with temozolomide and radiation therapy in treating patients with newly diagnosed glioblastoma	Drug: Atezolizumab Radiation: Radiation therapy Drug: Temozolomide	60	June 30, 2020
NCT03395587	Efficiency of vaccination with lysate-loaded dendritic cells in patients with newly diagnosed glioblastoma	Biological: Autologous, tumor lysate-loaded, mature dendritic cells (DC) Drug: Standard therapy	136	September 6, 2022
NCT03158389	NCT neuro master match–N^2^M^2^ (NOA-20)	Drug: APG101 Drug: Alectinib Drug: Idasanutlin (and 4 more.)	350	September 30, 2023
NCT03532295	INCMGA00012 and epacadostat in combination with radiation and bevacizumab in patients with recurrent gliomas	Drug: Epacadostat Drug: Bevacizumab Radiation: Radiation therapy Procedure: Peripheral blood draw	55	April 30, 2023
NCT03866109	A phase I/IIa study evaluating temferon in patients with glioblastoma & unmethylated MGMT	Drug: Temferon	21	December 2022
NCT03899857	Pembrolizumab for newly diagnosed glioblastoma	Drug: Pembrolizumab	56	December 2022
NCT01204684	Dendritic cell vaccine for patients with brain tumors	Biological: Autologous tumor lysate-pulsed DC vaccination Biological: Tumor lysate-pulsed DC vaccination+0.2% resiquimod Biological: Tumor-lysate pulsed DC vaccination +adjuvant polyICLC	60	January 31, 2021
NCT02968940	Avelumab with hypofractionated radiation therapy in adults with isocitrate dehydrogenase (IDH) mutant glioblastoma	Biological: Avelumab Radiation: Hypofractionated radiation therapy (HFRT)	43	April 2020
NCT02336165	Phase 2 Study of Durvalumab (MEDI4736) in Patients With Glioblastoma	Drug: Durvalumab Radiation: Standard radiotherapy Biological: Bevacizumab	159	November 2018
NCT04102436	Non-viral TCR gene therapy	Drug: Fludarabine Drug: Cyclophosphamide Drug: aldesleukin Biological: Sleeping Beauty Transposed PBL	210	December 31, 2028
NCT03412877	Administration of autologous T-cells genetically engineered to express T-cell receptors reactive against mutated neoantigens in people with metastatic cancer	Drug: Cyclophosphamide Drug: Fludarabine Drug: Aldesleukin (and 2 more.)	270	March 23, 2027
NCT02794883	Tremelimumab and durvalumab in combination or alone in treating patients with recurrent malignant glioma	Biological: Durvalumab Other: Laboratory Biomarker Analysis Procedure: Surgical Procedure Biological: Tremelimumab	36	December 2019
NCT03382977	Study to evaluate safety, tolerability, and optimal dose of candidate GBM vaccine VBI-1901 in recurrent GBM subjects	Biological: VBI-1901	38	October 2020
NCT03382977	Study to evaluate safety, tolerability, and optimal dose of candidate GBM vaccine VBI-1901 in recurrent GBM subjects	Biological: DNX-2401 Biological: Pembrolizumab	49	December 2020
**Phase I clinical trials**				
NCT02649582	Adjuvant dendritic cell-immunotherapy plus temozolomide in glioblastoma patients	Biological: Dendritic cell vaccine plus temozolomide chemotherapy	20	December 2020
NCT04165941	Novel gamma-delta γδ T cell therapy for treatment of patients with newly diagnosed glioblastoma	Biological: DRI cell therapy	12	January 2022
NCT03961971	Trial of anti-tim-3 in combination with anti-PD-1 and SRS in recurrent GBM	Drug: MBG453	15	February 2022
NCT03426891	Pembrolizumab and vorinostat combined with temozolomide for newly diagnosed glioblastoma	Drug: Pembrolizumab Drug: Vorinostat Drug: Temozolomide Radiation: Radiotherapy	32	April 2021
NCT02208362	Genetically modified T-cells in treating patients with recurrent or refractory malignant glioma	Biological: IL13Rα2-specific, hinge-optimized, 41BB-costimulatory CAR/truncated CD19-expressing Autologous T lymphocytes Other: Laboratory biomarker analysis Other: Quality-of-life assessment (and 5 more.)	92	May 2020
NCT04323046	Immunotherapy (nivolumab and ipilimumab) before and after surgery for the treatment of recurrent or progressive high grade glioma in children and young adults	Biological: Ipilimumab Biological: Nivolumab Drug: Placebo Administration (and 2 more.)	45	March 1, 2022
NCT04047706	Nivolumab, BMS-986205, and radiation therapy with or without temozolomide in treating patients with newly diagnosed glioblastoma	Biological: IDO1 inhibitor BMS-986205 Biological: nivolumab Radiation: Radiation Therapy Drug: Temozolomide	30	June 9, 2022
NCT04201873	Pembrolizumab and a vaccine (ATL-DC) for the treatment of surgically accessible recurrent glioblastoma	Biological: Dendritic cell tumor cell lysate vaccine Biological: Pembrolizumab Other: Placebo Administration Drug: Poly ICLC	40	August 1, 2024
NCT04003649	IL13Ralpha2-targeted chimeric antigen receptor (CAR) T cells with or without nivolumab and ipilimumab in treating patients with recurrent or refractory glioblastoma	Biological: IL13Ralpha2-specific Hinge-optimized 4-1BB-co-stimulatory CAR/Truncated CD19-expressing autologous TN/MEM cells Biological: Ipilimumab Biological: Nivolumab (and 2 more.)	60	January 22, 2022
NCT03714334	DNX-2440 oncolytic adenovirus for recurrent glioblastoma	Drug: DNX-2440 injection	24	April 16, 2022
NCT02852655	A pilot surgical trial to evaluate early immunologic pharmacodynamic parameters for The PD-1 checkpoint inhibitor, pembrolizumab (MK-3475), in patients with surgically accessible recurrent/progressive glioblastoma	Drug: MK-3475	35	March 28, 2018
NCT04270461	NKG2D-based CAR T-cells immunotherapy for patient with r/r NKG2DL+ solid tumors	Biological: NKG2D-based CAR T-cells	10	December 1, 2022
NCT03491683	INO-5401 and INO-9012 delivered by electroporation (EP) in combination with cemiplimab (REGN2810) in newly-diagnosed glioblastoma (GBM)	Biological: INO-5401 Biological: INO-9012 Biological: Cemiplimab (and 2 more.)	52	January 18, 2021
NCT03174197	Atezolizumab in Combination with temozolomide and radiation therapy in treating patients with newly diagnosed glioblastoma	Drug: Atezolizumab Radiation: Radiation Therapy Drug: Temozolomide	60	June 30, 2020
NCT03389230	Memory-enriched T cells in treating patients with recurrent or refractory grade III-IV glioma	Biological: CD19CAR-CD28-CD3zeta-EGFRt-expressing Tcm-enriched T-lymphocytes Biological: CD19CAR-CD28-CD3zeta-EGFRt-expressing Tn/mem-enriched T-lymphocytes Other: Laboratory Biomarker Analysis Procedure: Leukapheresis	42	June 14, 2021
NCT03344250	Phase I EGFR BATs in newly diagnosed glioblastoma	Drug: EGFR BATs with TMZ following SOC RT/TMZ Drug: Weekly EGFR BATs following SOC RT/TMZ	18	October 1, 2020
NCT03158389	NCT neuro master match–N^2^M^2^ (NOA-20)	Drug: APG101 Drug: Alectinib Drug: Idasanutlin (and 4 more.)	350	September 30, 2023
NCT03866109	A phase I/IIa study evaluating temferon in patients with glioblastoma & unmethylated MGMT	Drug: temFeron	21	December 2022
NCT03392545	Combination of immunization and radiotherapy for malignant gliomas (InSituVac1)	Combination product: Combined immune adjuvants and radiation	30	April 1, 2020
NCT03341806	Avelumab with laser interstitial therapy for recurrent glioblastoma	Drug: Avelumab Combination Product: MRI-guided LITT therapy	30	September 2020
NCT02062827	Genetically engineered HSV-1 phase 1 study for the treatment of recurrent malignant glioma	Biological: M032 (NSC 733972)	36	September 2020
NCT03223103	Safety and immunogenicity of personalized genomic vaccine and tumor treating fields (TTFields) to treat glioblastoma	Drug: Poly-ICLC Device: Tumor Treating Fields Biological: Peptides	20	May 22, 2020
NCT02766699	A study to evaluate the safety, tolerability and immunogenicity of EGFR(V)-EDV-dox in subjects with recurrent glioblastoma multiforme (GBM)	Drug: EGFR(V)-EDV-Dox	20	December 2019
NCT03619239	Dose-escalation study to evaluate the safety and tolerability of GX-I7 in patients with glioblastoma	Drug: GX-I7	15	January 31, 2021
NCT02010606	Phase I study of a dendritic cell vaccine for patients with either newly diagnosed or recurrent glioblastoma	Biological: Dendritic cell vaccination, in addition to standard temozolomide chemotherapy and involved field radiation therapy Biological: Dendritic cell vaccination, with optional bevacizumab treatment for patients previously treated with bevacizumab	39	April 2020
NCT02502708	Study of the IDO Pathway inhibitor, indoximod, and temozolomide for pediatric patients with progressive primary malignant brain tumors	Drug: Indoximod Drug: Temozolomide Radiation: Conformal radiation (and 2 more.)	81	December 12, 2019
NCT03382977	Study to evaluate safety, tolerability, and optimal dose of candidate GBM vaccine VBI-1901 in recurrent GBM subjects	Biological: VBI-1901	38	October 2020
NCT03043391	Phase 1b study PVSRIPO for recurrent malignant glioma in children	Biological: Polio/Rhinovirus Recombinant (PVSRIPO)	12	July 1, 2020
NCT03576612	GMCI, nivolumab, and radiation therapy in treating patients with newly diagnosed high-grade gliomas	Biological: AdV-tk Drug: Valacyclovir Radiation: Radiation (and 3 more.)	36	February 28, 2021
NCT03657576	Trial of C134 in patients with recurrent GBM	Biological: C134	24	September 2022
NCT03152318	A study of the treatment of recurrent malignant glioma with rQNestin34.5v.2	Drug: rQNestin Drug: Cyclophosphamide Procedure: Stereotactic biopsy	108	July 2021
NCT03911388	HSV G207 in children with recurrent or refractory cerebellar brain tumors	Biological: G207	15	September 1, 2022
NCT02457845	HSV G207 alone or with a single radiation dose in children with progressive or recurrent supratentorial brain tumors	Biological: G207	18	October 2020
NCT00634231	A phase I study of AdV-tk + prodrug therapy in combination with radiation therapy for pediatric brain tumors	Biological: AdV-tk Drug: Valacyclovir Radiation: Radiation	12	December 2015

## Author Contributions

All authors listed have made a substantial, direct and intellectual contribution to the work, and approved it for publication.

## Conflict of Interest

The authors declare that the research was conducted in the absence of any commercial or financial relationships that could be construed as a potential conflict of interest.

## References

[1] Machler P Wyss MT Elsayed M Stobart J Gutierrez R Von Faber-Castell A. *In vivo* evidence for a lactate gradient from astrocytes to neurons. Cell Metab. (2016) 23:94–102. 10.1016/j.cmet.2015.10.010 26698914

[2] Daubon T Leon C Clarke K Andrique L Salabert L Darbo E. Deciphering the complex role of thrombospondin-1 in glioblastoma development. Nat Commun. (2019) 10:1146. 10.1038/s41467-019-08480-y 30850588 PMC6408502

[3] Patel AP Tirosh I Trombetta JJ Shalek AK Gillespie SM Wakimoto H. Single-cell RNA-seq highlights intratumoral heterogeneity in primary glioblastoma. Science. (2014) 344:1396–401. 10.1126/science.1254257 24925914 PMC4123637

[4] Neftel C Laffy J Filbin MG Hara T Shore ME Rahme GJ. An integrative model of cellular states, plasticity, and genetics for glioblastoma. Cell. (2019) 178:835–49 e821. 10.1016/j.cell.2019.06.024 31327527 PMC6703186

[5] Iliff JJ Wang M Liao Y Plogg BA Peng W Gundersen GA. A paravascular pathway facilitates CSF flow through the brain parenchyma and the clearance of interstitial solutes, including amyloid beta. Sci Transl Med. (2012) 4:147ra111. 10.1126/scitranslmed.3003748 22896675 PMC3551275

[6] Aspelund A Antila S Proulx ST Karlsen TV Karaman S Detmar M. A dural lymphatic vascular system that drains brain interstitial fluid and macromolecules. J Exp Med. (2015) 212:991–9. 10.1084/jem.20142290 26077718 PMC4493418

[7] Louveau A Harris TH Kipnis J. Revisiting the mechanisms of CNS immune privilege. Trends Immunol. (2015) 36:569–77. 10.1016/j.it.2015.08.006 26431936 PMC4593064

[8] Medawar PB Immunity to homologous grafted skin; the fate of skin homografts transplanted to the brain, to subcutaneous tissue, and to the anterior chamber of the eye. Br J Exp Pathol. (1948) 29:58–69. 18865105 PMC2073079

[9] Andersson PB Perry VH Gordon S. The acute inflammatory response to lipopolysaccharide in CNS parenchyma differs from that in other body tissues. Neuroscience. (1992) 48:169–86. 10.1016/0306-4522(92)90347-5 1584421

[10] Locatelli G Wortge S Buch T Ingold B Frommer F Sobottka B. Primary oligodendrocyte death does not elicit anti-CNS immunity. Nat Neurosci. (2012) 15:543–50. 10.1038/nn.3062 22366759

[11] Ransohoff RM Brown MA. Innate immunity in the central nervous system. J Clin Invest. (2012) 122:1164–71. 10.1172/JCI58644 22466658 PMC3314450

[12] Mason DW Charlton HM Jones AJ Lavy CB Puklavec M Simmonds SJ. The fate of allogeneic and xenogeneic neuronal tissue transplanted into the third ventricle of rodents. Neuroscience. (1986) 19:685–94. 10.1016/0306-4522(86)90292-7 3796814

[13] Nicholas MK Antel JP Stefansson K Arnason BG. Rejection of fetal neocortical neural transplants by H-2 incompatible mice. J Immunol. (1987) 139:2275–83. 3309054

[14] Mrdjen D Pavlovic A Hartmann FJ Schreiner B Utz SG Leung BP High-dimensional single-cell mapping of central nervous system immune cells reveals distinct myeloid subsets in health, aging, and disease. Immunity. (2018) 48:599 10.1016/j.immuni.2018.01.011 29562204

[15] Sankowski R Bottcher C Masuda T Geirsdottir L Sagar Sindram E Seredenina T. Mapping microglia states in the human brain through the integration of high-dimensional techniques. Nat Neurosci. (2019) 22:2098–110. 10.1038/s41593-019-0532-y 31740814

[16] Mohammad MG Tsai VW Ruitenberg MJ Hassanpour M Li H Hart PH. Immune cell trafficking from the brain maintains CNS immune tolerance. J Clin Invest. (2014) 124:1228–41. 10.1172/JCI71544 24569378 PMC3934177

[17] Walsh JT Hendrix S Boato F Smirnov I Zheng J Lukens JR MHCII-independent CD4+ T cells protect injured CNS neurons via IL-4. J Clin Invest. (2015) 125:2547 10.1172/JCI76210 PMC4497772 25938785

[18] Louis DN Ohgaki H Wiestler OD Cavenee WK Burger PC Jouvet A. The 2007 WHO classification of tumours of the central nervous system. Acta Neuropathol. (2007) 114:97–109. 10.1007/s00401-007-0243-4 17618441 PMC1929165

[19] Verhaak RG Hoadley KA Purdom E Wang V Qi Y Wilkerson MD. Integrated genomic analysis identifies clinically relevant subtypes of glioblastoma characterized by abnormalities in PDGFRA, IDH1, EGFR, and NF1. Cancer Cell. (2010) 17:98–110. 10.1016/j.ccr.2009.12.020 20129251 PMC2818769

[20] Wang Q Hu B Hu X Kim H Squatrito M Scarpace L. Tumor evolution of glioma-intrinsic gene expression subtypes associates with immunological changes in the microenvironment. Cancer Cell. (2017) 32:42–56 e46. 10.1016/j.ccell.2017.06.003 28697342 PMC5599156

[21] Brennan CW Verhaak RG Mckenna A Campos B Noushmehr H Salama SR. The somatic genomic landscape of glioblastoma. Cell. (2013) 155:462–77. 10.1016/j.cell.2013.09.034 24120142 PMC3910500

[22] Frattini V Trifonov V Chan JM Castano A Lia M Abate F. The integrated landscape of driver genomic alterations in glioblastoma. Nat Genet. (2013) 45:1141–9. 10.1038/ng.2734 23917401 PMC3799953

[23] Eckel-Passow JE Lachance DH Molinaro AM Walsh KM Decker PA Sicotte H. Glioma groups based on 1p/19q, IDH, and TERT promoter mutations in tumors. N Engl J Med. (2015) 372:2499–508. 10.1056/NEJMoa1407279 26061753 PMC4489704

[24] Suzuki H Aoki K Chiba K Sato Y Shiozawa Y Shiraishi Y. Mutational landscape and clonal architecture in grade II and III gliomas. Nat Genet. (2015) 47:458–68. 10.1038/ng.3273 25848751

[25] Cancer Genome Atlas Research N Brat DJ Verhaak RG Aldape KD Yung WK Salama SR Comprehensive, integrative genomic analysis of diffuse lower-grade gliomas. N Engl J Med. (2015) 372:2481–98. 10.1056/NEJMoa1402121 26061751 PMC4530011

[26] Dewitt JC Mock A Louis DN. The 2016 WHO classification of central nervous system tumors: what neurologists need to know. Curr Opin Neurol. (2017) 30:643–9. 10.1097/WCO.0000000000000490 28901970

[27] Khuong-Quang DA Buczkowicz P Rakopoulos P Liu XY Fontebasso AM Bouffet E. K27M mutation in histone H3.3 defines clinically and biologically distinct subgroups of pediatric diffuse intrinsic pontine gliomas. Acta Neuropathol. (2012) 124:439–47. 10.1007/s00401-012-0998-0 22661320 PMC3422615

[28] Martinez-Lage M Lynch TM Bi Y Cocito C Way GP Pal S. Immune landscapes associated with different glioblastoma molecular subtypes. Acta Neuropathol Commun. (2019) 7:203. 10.1186/s40478-019-0803-6 31815646 PMC6902522

[29] Doucette T Rao G Rao A Shen L Aldape K Wei J. Immune heterogeneity of glioblastoma subtypes: extrapolation from the cancer genome atlas. Cancer Immunol Res. (2013) 1:112–22. 10.1158/2326-6066.CIR-13-0028 24409449 PMC3881271

[30] Newman AM Liu CL Green MR Gentles AJ Feng W Xu Y. Robust enumeration of cell subsets from tissue expression profiles. Nat Methods. (2015) 12:453–7. 10.1038/nmeth.3337 25822800 PMC4739640

[31] Ceccarelli M Barthel FP Malta TM Sabedot TS Salama SR Murray BA. Molecular profiling reveals biologically discrete subsets and pathways of progression in diffuse glioma. Cell. (2016) 164:550–63. 10.1016/j.cell.2015.12.028 26824661 PMC4754110

[32] Close HJ Stead LF Nsengimana J Reilly KA Droop A Wurdak H. Expression profiling of single cells and patient cohorts identifies multiple immunosuppressive pathways and an altered NK cell phenotype in glioblastoma. Clin Exp Immunol. (2020) 200:33–44. 10.1111/cei.13403 31784984 PMC7066386

[33] Mohme M Schliffke S Maire CL Runger A Glau L Mende KC. Immunophenotyping of newly diagnosed and recurrent glioblastoma defines distinct immune exhaustion profiles in peripheral and tumor-infiltrating lymphocytes. Clin Cancer Res. (2018) 24:4187–200. 10.1158/1078-0432.CCR-17-2617 29444930

[34] Denardo DG Ruffell B. Macrophages as regulators of tumour immunity and immunotherapy. Nat Rev Immunol. (2019) 19:369–82. 10.1038/s41577-019-0127-6 30718830 PMC7339861

[35] Cassetta L Pollard JW. Targeting macrophages: therapeutic approaches in cancer. Nat Rev Drug Discov. (2018) 17:887–904. 10.1038/nrd.2018.169 30361552

[36] Feng M Jiang W Kim BYS Zhang CC Fu YX Weissman IL. Phagocytosis checkpoints as new targets for cancer immunotherapy. Nat Rev Cancer. (2019) 19:568–86. 10.1038/s41568-019-0183-z 31462760 PMC7002027

[37] Butowski N Colman H De Groot JF Omuro AM Nayak L Wen PY. Orally administered colony stimulating factor 1 receptor inhibitor PLX3397 in recurrent glioblastoma: an Ivy Foundation Early Phase Clinical Trials Consortium phase II study. Neuro Oncol. (2016) 18:557–64. 10.1093/neuonc/nov245 26449250 PMC4799682

[38] Bowman RL Klemm F Akkari L Pyonteck SM Sevenich L Quail DF. Macrophage ontogeny underlies differences in tumor-specific education in brain malignancies. Cell Rep. (2016) 17:2445–59. 10.1016/j.celrep.2016.10.052 27840052 PMC5450644

[39] Castro BA Flanigan P Jahangiri A Hoffman D Chen W Kuang R. Macrophage migration inhibitory factor downregulation: a novel mechanism of resistance to anti-angiogenic therapy. Oncogene. (2017) 36:3749–59. 10.1038/onc.2017.1 28218903 PMC5491354

[40] Lavin Y Winter D Blecher-Gonen R David E Keren-Shaul H Merad M. Tissue-resident macrophage enhancer landscapes are shaped by the local microenvironment. Cell. (2014) 159:1312–26. 10.1016/j.cell.2014.11.018 25480296 PMC4437213

[41] Muller S Kohanbash G Liu SJ Alvarado B Carrera D Bhaduri A. Single-cell profiling of human gliomas reveals macrophage ontogeny as a basis for regional differences in macrophage activation in the tumor microenvironment. Genome Biol. (2017) 18:234. 10.1186/s13059-017-1362-4 29262845 PMC5738907

[42] Hutter G Theruvath J Graef CM Zhang M Schoen MK Manz EM. Microglia are effector cells of CD47-SIRPalpha antiphagocytic axis disruption against glioblastoma. Proc Natl Acad Sci USA. (2019) 116:997–1006. 10.1073/pnas.1721434116 30602457 PMC6338872

[43] Chen Z Feng X Herting CJ Garcia VA Nie K Pong WW. Cellular and molecular identity of tumor-associated macrophages in glioblastoma. Cancer Res. (2017) 77:2266–78. 10.1158/0008-5472.CAN-16-2310 28235764 PMC5741820

[44] Chen Z Ross JL Hambardzumyan D. Intravital 2-photon imaging reveals distinct morphology and infiltrative properties of glioblastoma-associated macrophages. Proc Natl Acad Sci USA. (2019) 116:14254–9. 10.1073/pnas.1902366116 31235603 PMC6628659

[45] Couto M Coelho-Santos V Santos L Fontes-Ribeiro C Silva AP Gomes CMF. The interplay between glioblastoma and microglia cells leads to endothelial cell monolayer dysfunction via the interleukin-6-induced JAK2/STAT3 pathway. J Cell Physiol. (2019) 234:19750–60. 10.1002/jcp.28575 30937892

[46] Wang Q He Z Huang M Liu T Wang Y Xu H. Vascular niche IL-6 induces alternative macrophage activation in glioblastoma through HIF-2alpha. Nat Commun. (2018) 9:559. 10.1038/s41467-018-03050-0 29422647 PMC5805734

[47] Zhang Y Yu G Chu H Wang X Xiong L Cai G. Macrophage-associated PGK1 phosphorylation promotes aerobic glycolysis and tumorigenesis. Mol Cell. (2018) 71:201–15 e207. 10.1016/j.molcel.2018.06.023 30029001

[48] Takenaka MC Gabriely G Rothhammer V Mascanfroni ID Wheeler MA Chao CC Control of tumor-associated macrophages and T cells in glioblastoma via AHR and CD39. Nat Neurosci. (2019) 22:729–40. 10.1038/s41593-019-0370-y 30962630 PMC8052632

[49] Chen P Zhao D Li J Liang X Li J Chang A. Symbiotic macrophage-glioma cell interactions reveal synthetic lethality in PTEN-Null glioma. Cancer Cell. (2019) 35:868–84 e866. 10.1016/j.ccell.2019.05.003 31185211 PMC6561349

[50] Wei J Marisetty A Schrand B Gabrusiewicz K Hashimoto Y Ott M. Osteopontin mediates glioblastoma-associated macrophage infiltration and is a potential therapeutic target. J Clin Invest. (2019) 129:137–49. 10.1172/JCI121266 30307407 PMC6307970

[51] Kuppner MC Hamou MF Sawamura Y Bodmer S De Tribolet N. Inhibition of lymphocyte function by glioblastoma-derived transforming growth factor beta 2. J Neurosurg. (1989) 71:211–7. 10.3171/jns.1989.71.2.0211 2545842

[52] Jackson CM Kochel CM Nirschl CJ Durham NM Ruzevick J Alme A. Systemic tolerance mediated by melanoma brain tumors is reversible by radiotherapy and vaccination. Clin Cancer Res. (2016) 22:1161–72. 10.1158/1078-0432.CCR-15-1516 26490306 PMC4825863

[53] Bloch O Crane CA Kaur R Safaee M Rutkowski MJ Parsa AT. Gliomas promote immunosuppression through induction of B7-H1 expression in tumor-associated macrophages. Clin Cancer Res. (2013) 19:3165–75. 10.1158/1078-0432.CCR-12-3314 23613317 PMC3742575

[54] Parsa AT Waldron JS Panner A Crane CA Parney IF Barry JJ. Loss of tumor suppressor PTEN function increases B7-H1 expression and immunoresistance in glioma. Nat Med. (2007) 13:84–8. 10.1038/nm1517 17159987

[55] Wischhusen J Friese MA Mittelbronn M Meyermann R Weller M. HLA-E protects glioma cells from NKG2D-mediated immune responses *in vitro*: implications for immune escape *in vivo*. J Neuropathol Exp Neurol. (2005) 64:523–8. 10.1093/jnen/64.6.523 15977644

[56] Didenko VV Ngo HN Minchew C Baskin DS. Apoptosis of T lymphocytes invading glioblastomas multiforme: a possible tumor defense mechanism. J Neurosurg. (2002) 96:580–4. 10.3171/jns.2002.96.3.0580 11883844 PMC1853267

[57] Dusoswa SA Verhoeff J Abels E Mendez-Huergo SP Croci DO Kuijper LH. Glioblastomas exploit truncated O-linked glycans for local and distant immune modulation via the macrophage galactose-type lectin. Proc Natl Acad Sci USA. (2020) 117:3693–703. 10.1073/pnas.1907921117 32019882 PMC7035608

[58] Lai SW Lin HJ Liu YS Yang LY Lu DY. Monocarboxylate transporter 4 regulates glioblastoma motility and monocyte binding ability. Cancers. (2020) 12:380. 10.3390/cancers12020380 32045997 PMC7073205

[59] Brand A Singer K Koehl GE Kolitzus M Schoenhammer G Thiel A. LDHA-associated lactic acid production blunts tumor immunosurveillance by T and NK cells. Cell Metab. (2016) 24:657–71. 10.1016/j.cmet.2016.08.011 27641098

[60] Fischer K Hoffmann P Voelkl S Meidenbauer N Ammer J Edinger M. Inhibitory effect of tumor cell-derived lactic acid on human T cells. Blood. (2007) 109:3812–9. 10.1182/blood-2006-07-035972 17255361

[61] Silva LS Poschet G Nonnenmacher Y Becker HM Sapcariu S Gaupel AC. Branched-chain ketoacids secreted by glioblastoma cells via MCT1 modulate macrophage phenotype. EMBO Rep. (2017) 18:2172–85. 10.15252/embr.201744154 29066459 PMC5709768

[62] Reardon DA Brandes AA Omuro A Mulholland P Lim M Wick A. Effect of nivolumab vs bevacizumab in patients with recurrent glioblastoma: the CheckMate 143 phase 3 randomized clinical trial. JAMA Oncol. (2020) 6:1003–10. 10.1001/jamaoncol.2020.1024 32437507 PMC7243167

[63] Schalper KA Rodriguez-Ruiz ME Diez-Valle R Lopez-Janeiro A Porciuncula A Idoate MA. Neoadjuvant nivolumab modifies the tumor immune microenvironment in resectable glioblastoma. Nat Med. (2019) 25:470–6. 10.1038/s41591-018-0339-5 30742120

[64] Cloughesy TF Mochizuki AY Orpilla JR Hugo W Lee AH Davidson TB. Neoadjuvant anti-PD-1 immunotherapy promotes a survival benefit with intratumoral and systemic immune responses in recurrent glioblastoma. Nat Med. (2019) 25:477–86. 10.1038/s41591-018-0337-7 30742122 PMC6408961

[65] Maxwell JA Johnson SP Mclendon RE Lister DW Horne KS Rasheed A. Mismatch repair deficiency does not mediate clinical resistance to temozolomide in malignant glioma. Clin Cancer Res. (2008) 14:4859–68. 10.1158/1078-0432.CCR-07-4807 18676759 PMC2830553

[66] Bouffet E Larouche V Campbell BB Merico D De Borja R Aronson M. Immune checkpoint inhibition for hypermutant glioblastoma multiforme resulting from germline biallelic mismatch repair deficiency. J Clin Oncol. (2016) 34:2206–11. 10.1200/JCO.2016.66.6552 27001570

[67] Johanns TM Miller CA Dorward IG Tsien C Chang E Perry A. Immunogenomics of hypermutated glioblastoma: a patient with germline POLE deficiency treated with checkpoint blockade immunotherapy. Cancer Discov. (2016) 6:1230–6. 10.1158/2159-8290.CD-16-0575 27683556 PMC5140283

[68] Touat M Li YY Boynton AN Spurr LF Iorgulescu JB Bohrson CL. Mechanisms and therapeutic implications of hypermutation in gliomas. Nature. (2020) 580:517–23. 10.1038/s41586-020-2209-9 32322066 PMC8235024

[69] Yang C Austin F Richard H Idowu M Williamson V Sabato F. Lynch syndrome-associated ultra-hypermutated pediatric glioblastoma mimicking a constitutional mismatch repair deficiency syndrome. Cold Spring Harb Mol Case Stud. (2019) 5:a003863. 10.1101/mcs.a003863 31604779 PMC6824252

[70] Le DT Durham JN Smith KN Wang H Bartlett BR Aulakh LK. Mismatch repair deficiency predicts response of solid tumors to PD-1 blockade. Science. (2017) 357:409–13. 10.1126/science.aan6733 28596308 PMC5576142

[71] Galstyan A Markman JL Shatalova ES Chiechi A Korman AJ Patil R. Blood-brain barrier permeable nano immunoconjugates induce local immune responses for glioma therapy. Nat Commun. (2019) 10:3850. 10.1038/s41467-019-11719-3 31462642 PMC6713723

[72] Bateman ME Strong AL Gimble JM Bunnell BA. Concise review: using fat to fight disease: a systematic review of nonhomologous adipose-derived stromal/stem cell therapies. Stem Cells. (2018) 36:1311–28. 10.1002/stem.2847 29761573

[73] Weller M Butowski N Tran DD Recht LD Lim M Hirte H. Rindopepimut with temozolomide for patients with newly diagnosed, EGFRvIII-expressing glioblastoma (ACT IV): a randomised, double-blind, international phase 3 trial. Lancet Oncol. (2017) 18:1373–85. 10.1016/S1470-2045(17)30517-X 28844499

[74] Liau LM Ashkan K Tran DD Campian JL Trusheim JE Cobbs CS First results on survival from a large Phase 3 clinical trial of an autologous dendritic cell vaccine in newly diagnosed glioblastoma. J Transl Med. (2018) 16:142 10.1186/s12967-018-1552-1 29843811 PMC5975654

[75] Narita Y Arakawa Y Yamasaki F Nishikawa R Aoki T Kanamori M. A randomized, double-blind, phase III trial of personalized peptide vaccination for recurrent glioblastoma. Neuro Oncol. (2019) 21:348–59. 10.1093/neuonc/noy200 30500939 PMC6380422

[76] Hilf N Kuttruff-Coqui S Frenzel K Bukur V Stevanovic S Gouttefangeas C. Actively personalized vaccination trial for newly diagnosed glioblastoma. Nature. (2019) 565:240–5. 10.1038/s41586-018-0810-y 30568303

[77] Keskin DB Anandappa AJ Sun J Tirosh I Mathewson ND Li S. Neoantigen vaccine generates intratumoral T cell responses in phase Ib glioblastoma trial. Nature. (2019) 565:234–9. 10.1038/s41586-018-0792-9 30568305 PMC6546179

[78] Brown CE Alizadeh D Starr R Weng L Wagner JR Naranjo A. Regression of glioblastoma after chimeric antigen receptor T-cell therapy. N Engl J Med. (2016) 375:2561–9. 10.1056/NEJMoa1610497 28029927 PMC5390684

[79] O'rourke DM Nasrallah MP Desai A Melenhorst JJ Mansfield K Morrissette JJD. A single dose of peripherally infused EGFRvIII-directed CAR T cells mediates antigen loss and induces adaptive resistance in patients with recurrent glioblastoma. Sci Transl Med. (2017) 9:984. 10.1126/scitranslmed.aaa0984 28724573 PMC5762203

[80] Pellegatta S Savoldo B Di Ianni N Corbetta C Chen Y Patane M. Constitutive and TNFalpha-inducible expression of chondroitin sulfate proteoglycan 4 in glioblastoma and neurospheres: implications for CAR-T cell therapy. Sci Transl Med. (2018) 10:eaao2731. 10.1126/scitranslmed.aao2731 29491184 PMC8713441

[81] Krenciute G Prinzing BL Yi Z Wu MF Liu H Dotti G. Transgenic expression of IL15 improves antiglioma activity of IL13Ralpha2-CAR T cells but results in antigen loss variants. Cancer Immunol Res. (2017) 5:571–81. 10.1158/2326-6066.CIR-16-0376 28550091 PMC5746871

[82] Wang D Aguilar B Starr R Alizadeh D Brito A Sarkissian A. Glioblastoma-targeted CD4+ CAR T cells mediate superior antitumor activity. JCI Insight. (2018) 3. 10.1172/jci.insight.99048 29769444 PMC6012522

[83] Jin L Tao H Karachi A Long Y Hou AY Na M. CXCR1- or CXCR2-modified CAR T cells co-opt IL-8 for maximal antitumor efficacy in solid tumors. Nat Commun. (2019) 10:4016. 10.1038/s41467-019-11869-4 31488817 PMC6728370

[84] Choi BD Yu X Castano AP Bouffard AA Schmidts A Larson RC. CAR-T cells secreting BiTEs circumvent antigen escape without detectable toxicity. Nat Biotechnol. (2019) 37:1049–58. 10.1038/s41587-019-0192-1 31332324

[85] Han J Chu J Keung Chan W Zhang J Wang Y Cohen JB. CAR-engineered NK cells targeting wild-type EGFR and EGFRvIII enhance killing of glioblastoma and patient-derived glioblastoma stem cells. Sci Rep. (2015) 5:11483. 10.1038/srep11483 26155832 PMC4496728

[86] Nehama D Di Ianni N Musio S Du H Patane M Pollo B. B7-H3-redirected chimeric antigen receptor T cells target glioblastoma and neurospheres. EBioMedicine. (2019) 47:33–43. 10.1016/j.ebiom.2019.08.030 31466914 PMC6796553

[87] Tang X Zhao S Zhang Y Wang Y Zhang Z Yang M. B7-H3 as a novel CAR-T therapeutic target for glioblastoma. Mol Ther Oncolytics. (2019) 14:279–87. 10.1016/j.omto.2019.07.002 31485480 PMC6713854

[88] Burger MC Zhang C Harter PN Romanski A Strassheimer F Senft C. CAR-engineered NK cells for the treatment of glioblastoma: turning innate effectors into precision tools for cancer immunotherapy. Front Immunol. (2019) 10:2683. 10.3389/fimmu.2019.02683 31798595 PMC6868035

[89] Hegde M Mukherjee M Grada Z Pignata A Landi D Navai SA Tandem CAR T cells targeting HER2 and IL13Ralpha2 mitigate tumor antigen escape. J Clin Invest. (2019) 129:3464 10.1172/JCI131246 PMC6668664 31264975

[90] Shen L Li H Bin S Li P Chen J Gu H The efficacy of third generation antiHER2 chimeric antigen receptor T cells in combination with PD1 blockade against malignant glioblastoma cells. Oncol Rep. (2019) 42:1549–57. 10.3892/or.2019.7263 31524276

[91] Yi Z Prinzing BL Cao F Gottschalk S Krenciute G. Optimizing EphA2-CAR T cells for the adoptive immunotherapy of glioma. Mol Ther Methods Clin Dev. (2018) 9:70–80. 10.1016/j.omtm.2018.01.009 29552579 PMC5852415

[92] Ahmed N Brawley V Hegde M Bielamowicz K Kalra M Landi D. HER2-specific chimeric antigen receptor-modified virus-specific T cells for progressive glioblastoma: a phase 1 dose-escalation trial. JAMA Oncol. (2017) 3:1094–101. 10.1001/jamaoncol.2017.0184 28426845 PMC5747970

[93] Bielamowicz K Fousek K Byrd TT Samaha H Mukherjee M Aware N Trivalent CAR T cells overcome interpatient antigenic variability in glioblastoma. Neuro Oncol. (2018) 20:506–18. 10.1093/neuonc/nox182 29016929 PMC5909636

[94] Wang D Starr R Chang WC Aguilar B Alizadeh D Wright SL. Chlorotoxin-directed CAR T cells for specific and effective targeting of glioblastoma. Sci Transl Med. (2020) 12:eaaw2672. 10.1126/scitranslmed.aaw2672 32132216 PMC7500824

[95] Desjardins A Gromeier M Herndon JE 2nd Beaubier N Bolognesi DP Friedman AH. Recurrent glioblastoma treated with recombinant poliovirus. N Engl J Med. (2018) 379:150–61. 10.1056/NEJMoa1716435 29943666 PMC6065102

[96] Ji N Weng D Liu C Gu Z Chen S Guo Y. Adenovirus-mediated delivery of herpes simplex virus thymidine kinase administration improves outcome of recurrent high-grade glioma. Oncotarget. (2016) 7:4369–78. 10.18632/oncotarget.6737 26716896 PMC4826211

[97] Wheeler LA Manzanera AG Bell SD Cavaliere R Mcgregor JM Grecula JC. Phase II multicenter study of gene-mediated cytotoxic immunotherapy as adjuvant to surgical resection for newly diagnosed malignant glioma. Neuro Oncol. (2016) 18:1137–45. 10.1093/neuonc/now002 26843484 PMC4933478

[98] Xu B Ma R Russell L Yoo JY Han J Cui H. An oncolytic herpesvirus expressing E-cadherin improves survival in mouse models of glioblastoma. Nat Biotechnol. (2018) 37:45–54. 10.1038/nbt.4302 30475349 PMC6535376

[99] Zhu Z Mesci P Bernatchez JA Gimple RC Wang X Schafer ST. Zika virus targets glioblastoma stem cells through a SOX2-Integrin alphavbeta5 Axis. Cell Stem Cell. (2020) 26:187–204 e110. 10.1016/j.stem.2019.11.016 31956038 PMC9628766

[100] Saha D Martuza RL Rabkin SD. Curing glioblastoma: oncolytic HSV-IL12 and checkpoint blockade. Oncoscience. (2017) 4:67–9. 10.18632/oncoscience.359 28966936 PMC5616196

[101] Gholamin S Mitra SS Feroze AH Liu J Kahn SA Zhang M. Disrupting the CD47-SIRPalpha anti-phagocytic axis by a humanized anti-CD47 antibody is an efficacious treatment for malignant pediatric brain tumors. Sci Transl Med. (2017) 9:eaaf2968. 10.1126/scitranslmed.aaf2968 28298418

[102] Von Roemeling CA Wang Y Qie Y Yuan H Zhao H Liu X. Therapeutic modulation of phagocytosis in glioblastoma can activate both innate and adaptive antitumour immunity. Nat Commun. (2020) 11:1508. 10.1038/s41467-020-15129-8 32198351 PMC7083893

[103] Buonfiglioli A Efe IE Guneykaya D Ivanov A Huang Y Orlowski E. let-7 MicroRNAs regulate microglial function and suppress glioma growth through toll-like receptor 7. Cell Rep. (2019) 29:3460–71. e3467. 10.1016/j.celrep.2019.11.029 31825829

[104] Zhang F Parayath NN Ene CI Stephan SB Koehne AL Coon ME. Genetic programming of macrophages to perform anti-tumor functions using targeted mRNA nanocarriers. Nat Commun. (2019) 10:3974. 10.1038/s41467-019-11911-5 31481662 PMC6722139

